# The Tentacular Strike Behavior in Squid: Functional Interdependency of Morphology and Predatory Behaviors During Ontogeny

**DOI:** 10.3389/fphys.2019.01558

**Published:** 2019-12-31

**Authors:** Erica A. G. Vidal, Bianca Salvador

**Affiliations:** Center for Marine Studies, Federal University of Parana, Pontal do Paraná, Brazil

**Keywords:** arm crown, cephalopod, *Doryteuthis opalescens*, feeding behavior, paralarvae, prey types, tentacles

## Abstract

This study examines the relationship between morphology and predatory behaviors to evaluate the ontogeny of the specialized tentacular strike (TS) in *Doryteuthis opalescens* squid reared under laboratory conditions [hatching to 80 day-old; 2–16 mm mantle length (ML)]. Ontogenetic morphological changes in the arm-crown and the role played by the arms and tentacles during predatory behavior was correlated with prey types captured and revealed interconnected morphological and behavior traits that enabled paralarvae to perform the TS. Hatchlings have a poorly developed arm-crown and tentacles that resemble and function as arms, in which tentacular clubs (suckerfull non-contractile portion) and stalks (suckerless contractile portion) have not yet formed. Only a basic attack (BA) behavior was observed, involving arms and tentacles, which were not ejected during prey capture. A more elaborated behavior, the arm-net (AN) was first employed by 30 day-old (>4.7 mm ML) paralarvae, in which the tentacles were eject down, but not toward the prey. The TS was first observed in 40–50 day-old (6.7–7.8 mm ML) squid, which stay stationary by sustainable swimming prior to ejecting the tentacles toward the prey. Thus, the ability to perform sustainable swimming and acquisition of swimming coordination (schooling behavior) are prerequisites for the expression of the TS. The arms played the same roles after prey was captured: hold, subdue and manipulate the prey, while the actions performed by the tentacles truly defined each behavior. Prey size captured increased with increasing squid size. Morphometric data showed that hatchlings have little ability of elongating their tentacles, but this ability increases significantly with size. Squid older than 40 days could elongate their tentacles up to 61% of their ML, whereas early paralarvae 13% on average. Paralarvae were frequently observed elongating and contracting their tentacles, while not attempting to capture prey, which could perhaps serve to adjust muscle activity and development, while specializations for the strike – stalks, clubs, muscle fibers, arm-crown and swimming coordination – are still being developed. The expression of the TS is constrained by development in early paralarvae as it involves interdependency of morphology and behavior and as such, represents a major developmental milestone in the early life history of squid.

## Introduction

The arm crown of Decapodiform cephalopods consists of ten appendages enclosing the mouth. Eight of these appendages – the arms – possess suckers along their entire length, while the other two appendages – the tentacles – possess suckers only at the distal portion. The arms and tentacles of adult squid differ fundamentally in their function, ultrastructure, and behavior (Kier, 1991; Boletzky, 1993). The primary function of the arms is prey capture and manipulation, but they are also involved in behavioral displays, locomotion stabilization, and reproduction. The tentacles are specialized for prey capture and possess a unique capacity for fast elongation (Kier, 1996, 2016).

During the tentacular strike (TS) predatory behavior, the stalks (tentacles suckerless proximal portion) are elongated so the clubs (tentacles suckerfull distal portion) make the first contact with prey and attach to it. Then the stalks are immediately contracted to bring the prey within reach of the arms, which subdue and manipulate the prey during ingestion. The tentacular stalk muscles are capable of an extremely rapid extension that reaches the prey in a straight line in a remarkable 20–40 ms, with maximum stalk extension velocities reaching over 2 ms^–1^ and peak accelerations of nearly 250 ms^–2^ (Kier, 1982; Kier and van Leeuwen, 1997).

The ultrastructure of the transverse muscle cells of the tentaclular stalk is different from all other cephalopod musculature. It shows cross-striation, short-sarcomere, and thick filaments, which are specializations that enable the fast elongation of the tentacular stalks to reach the prey during the strike (Kier, 1992, 1996). The transverse muscle fibers of the arms instead are obliquely striated and responsible for slower movements of bending and twisting to subdue and manipulate the prey (Kier and Schachat, 1992).

The musculoskeletal system of arms and tentacles in squid function as a muscular-hydrostatic mechanism in which the musculature itself serves as the hydrostatic fluid. As such, they are incompressible at physiological pressures and constant in volume. Thus, a decrease in one dimension will result in an increase in another (Kier and Smith, 1985). During the strike, elongation of the stalk is caused by the contraction of the muscle cells of the transverse muscle mass, which decreases in diameter. After prey is attached to the club’s suckers, the stalk is shortened by contraction of the longitudinal muscle bundles, causing the elongation of the transverse muscle cells (Kier, 1991).

The specialization of tentacles transverse muscle fibers for fast contraction allude to the evolutionary history of coleoid cephalopods (Donovan, 1977; Boletzky, 1993; Kier and van Leeuwen, 1997). From the original five pairs of arms in the line that gave rise to the Decapodiform cephalopods, the tentacles have evolved through modifications of the fourth pair of arms in the ancestral coleoid. In this regard, it was suggested that the transverse muscle of arms and tentacles are homologous, and the cross-striated muscle fiber have evolved through a reorganization of the obliquely striated muscle cells (Kier, 1985, 1991).

In the present context, it is of significance that the tentacles of loliginid squid hatchlings do not possess the adult cross-striated ultrastructure responsible for fast contraction to perform the strike (Kier, 1996; Kier and van Leeuwen, 1997). This was demonstrated in a comprehensive study correlating predatory behaviors with tentacles muscle fiber differentiation in *Sepioteuthis lessoniana* (Kier, 1996). Thus, the predatory behavior of paralarvae and adults must differ fundamentally and paralarvae foraging techniques must evolve until they can perform the TS. These differences must arise out of constraints on development, which seems to be the most important factor influencing paralarvae foraging behavior and ecological niche.

Evaluation of the sequential underlying factors responsible for the expression of the TS can offer important insights into the way this specialized behavior is ultimately constraint in paralarvae. Indeed, the means by which specializations arise in ontogeny may provide clues on how developmental constraints impose behavioral adaptations (Boletzky, 1997). This in turn, is paramount for a better understanding of paralarvae adaptive foraging strategies and its evolutionary and ecological consequences.

Laboratory studies have been the basis for much of what is known about predatory behavior in squid paralarvae. The ontogeny of copepod predation in laboratory reared *Doryteuthis opalescens* have shown that paralarvae capture their prey using the arms and the TS was only observed in four weeks old squid (Chen et al., 1996).

Upon hatching, squid paralarvae have bell shape, limited swimming and behavioral abilities (Chen et al., 1996; Vidal et al., 2018) and an underdeveloped arm-crown and beak (Franco-Santos and Vidal, 2014). In contrast, during their first month of life, squid undergo foremost morphological, behavioral, and ecological changes, leading to enhanced swimming performance and cognitive abilities, which make them competent to swim in schools and control their distribution (Vidal et al., 2018). This coincides with the end of the paralarval dispersive phase and the transition from plankton to nekton, representing a shift in paralarvae physiological ecology (Vidal et al., 2018). In concert with these major developmental landmarks that squid goes through during early ontogeny, their predatory behavior and functional morphology changes.

The present study integrates behavioral observations with morphological and morphometric data to obtain a detailed understanding of tentacular kinematics and morphology, while also evaluating the role played by the arms and tentacles during predatory behavior in *D. opalescens* reared under laboratory conditions from hatching to 80 days of age. Importantly, comprehensive available information on feeding behavior and prey types, growth, body and beak morphology, and swimming abilities for *D. opalescens* (Chen et al., 1996; Vidal et al., 2002, 2018; Franco-Santos and Vidal, 2014; Vidal and Boletzky, 2014) provides the unique opportunity to encompass several levels of knowledge in examining how predatory behavior relates to key events during ontogeny. Thus, the aims of this study are to evaluate the ontogeny of predatory behavior, particularly the TS behavior, and its relationship with morphological development. In addition, we correlated arm-crown morphology and predatory behaviors with the prey types captured by paralarvae during ontogeny.

## Materials and Methods

### Rearing of Paralarvae and Video Recordings of Predatory Behavior

*Doryteuthis opalescens* eggs were collected by SCUBA divers in Monterey Bay (36°60′N, 121°80′W) and Southern California (34°7′N, 119°05′W), United States. Eggs were transferred to the National Resource Center for Cephalopods, University of Texas Medical Branch, Galveston, TX, and upon hatching paralarvae were raised up to 80 days after hatching on a closed recirculating system. Detailed information on rearing of eggs and paralarvae are found in Vidal et al. (2002).

Paralarvae ranging in size from 2 to 13 mm mantle length (ML) were filmed at 0, 1, 2, 6, 10, 11, 13, 14, 17, 20, 30, 40, 50, 55, and 60 days of age. Mean ML for paralarvae of these ages (0 = 2.65 mm ML, 6 = 2.7 mm ML, 14 = 3.8 mm ML, 20 = 4.1 mm ML, 30 = 4.7 mm ML, 40 = 6.7 mm ML, 55 = 7.8 mm ML, 60 = 9.8 mm ML) were obtained from Vidal et al. (2018) as filming used in the present study were conducted simultaneously with the experiments reported in that particular study. A round aquarium (7 cm H, 12 cm diameter and holding a center core of 9 cm) was constructed as a miniature of the large rearing tanks, in which filming was performed from above (dorsal perspective). A rectangular aquarium (14 cm L, 15 cm H, 3 cm W) was used to film early paralarvae (0–30 day-old), and a larger rectangular aquarium (30 cm L, 20cm H, 6.5 cm W) was used to film larger paralarvae (40–60 day-old). In the rectangular aquaria, filming was performed from the side (lateral perspective), that in combination with the dorsal view (round aquarium), resulted in a three-dimensional understanding of predatory behaviors. Conditions of the large holding tanks were reproduced in the small aquaria (i.e., temperature was kept at 16°C, a small current was generated to homogeneously distribute the paralarvae and prey, and the walls were covered on the outside with a flat-black plastic to enhance prey contrast and to reduce reflectance).

From 8 to 20 paralarvae of each age were collected at random and transferred from the large rearing tanks (200 L) to the small aquaria from 4 to 5 h prior to filming. They were fed *ad libitum* on *Artemia* spp. nauplii enriched with SUPER SELCO (INVE), juvenile and adults mysid shrimp (*Americamysis almyra*) and wild zooplankton, composed mainly by copepods (adults and copepodits), but containing also a wide variety of other zooplankton organism such as crustacean zoeae and myses, Cladocera, Cirripedia nauplii, etc. Feeding a variety of prey types and sizes is important to maintain high survival rates, which during the first 60 days of rearing ranged from 42 to 60% (Vidal et al., 2002). More information on live prey composition, size and developmental stages offered to paralarvae and juveniles during rearing can be found in Vidal et al. (2002) and Vidal and Boletzky (2014).

A Sony CCD-TR930 Digital Hi8 camcorder fitted with #1.5 close–up lens and operating at 30 frames s^–1^ was used to film paralarvae and record their predatory behavior. Frame-by-frame analyses were performed with a Sony CVD-1000 Hi8 editing deck. The camera was mounted next to the aquaria at a 90° angle; the frame of view for filming was 3.6 × 3.6 cm. A thin ruler was positioned inside the aquaria to set the scale for each image and distance calibration was performed prior to each filming session. The camera was set to operate in manual mode and the focus was adjusted to the ruler with a focal distance of 1–3 cm in toward the center of the aquaria. The autofocus and zoom functions of the camera were turned off and the lens aperture was locked to maintain a constant depth of field. Paralarvae were videotaped when they were in focus for the small depth of field. The erros resuting from the positioning of the paralarvae along the optical axis were estimated to be below 15% for hatchlings and decreased as paralarvae increased in size.

To evaluate the role played by the arms and tentacles during prey capture paralarvae were filmed during attack attempts and prey capture sequences. Approximately 36 hrs of filming observations were analyzed frame-by-frame (from 2 to 2.5 h for each age filmed). There were over 500 predator-prey interactions, proportionally distributed among the ages evaluated, where the behavior of paralarvae toward a prey could be analyzed and the role played by each arm and the tentacles observed. During filming behavior, each prey captured by paralarvae was recorded and correlated with the predatory behavior employed. Behavior monitoring was performed through a TV set so that the paralarvae were not disturbed during filming. To obtain prey sizes, a sample (10–15 individuals) of the main prey types offered to paralarvae was taken from the zooplankton maintenance tanks and fixed in 4% formaldehyde–seawater for subsequent identification and size measurement under a dissecting stereomicroscope equipped with an ocular micrometer.

### Arm-Crown Morphological Development and Measurements of Tentacular Elongations

Morphological development of arms and tentacles of paralarvae and juveniles were observed in 30 specimens from 1 to 80 days of age (2–16 mm ML) at every 10 d interval through a dissecting stereomicroscope, when mean tentacles length (TL) was measured.

The measurements of tentacular elongations and ML from the same paralarva were performed on the public domain software NIH Image (version 1.61) using the images recorded. The ruler placed inside the aquaria provided a reference scale and length-values were stored in the Result Window of the NIH Image software. To ensure the accuracy and precision of measurements, TL was only measured when the squid were within a predefined distance and orientation to the camera, when their eyes were exactly parallel to the video camera and in focus. To obtain the length of the tentacles, it was necessary to use an external landmark, as the base of the tentacles is enclosed within the arm crown of paralarvae. Thus, the anterior margin of the lens of the eyes was used as this landmark following the methodology of Kier and van Leeuwen (1997). Measurements of fully elongated and contracted tentacles from the same individual were obtained from 8 to 20 squid of each age during the first 60 days of age. These measurements were obtained from filmed performed on the rectangular aquaria, when paralarvae were not attempting to capture prey. The contracted TL was measured as the distance between the anterior margin of the eye lens and the extremity of fully contracted tentacles and, the elongated TL as the distance between the anterior margin of the eye lens and the extremity of fully elongated tentacles.

The ability of paralarvae to elongate its tentacles was calculated as the length difference between fully elongated and contracted tentacles (tentacular elongation). The results were normalized by the ML and converted in percentage. The tentacular strain (ε) was calculated according to Kier and van Leeuwen (1997) as:

ε=(l-l)0/l,0

where *l* = final TL and *l*_0_ = initial TL.

### Data Analysis

To compare the relationship between contracted and elongated TL and ML, TL was log-transformed and the differences between regression slopes and intercepts of growth curves were tested by analysis of covariance (ANCOVA; Sokal and Rohlf, 1981). The validity of growth curves was accepted only when the slopes and intercepts between the regression lines of contracted and elongated tentacles and ML showed significant differences and when slopes were significantly different from zero (Sokal and Rohlf, 1981).

Differences in the percentage of tentacular elongation and in the tentacular strain between paralarvae of different ages were tested by analysis of variance (ANOVA). The age groups analyzed were 1–10, 14, 20, 30, 40, 55, and 60 day-old paralarvae. The first category (1–10) included length measurements taken from 1, 6, and 10 day-old paralarvae. Overall statistically significant differences between groups were reported by ANOVA and *post hoc* pairwise comparisons among all groups’ means were conducted with Tukey HSD test to specify which age groups were significantly different from each other. The data was fourth root (tentacular elongation) and square root (tentacular strain) transformed prior to analysis to satisfy normality assumptions.

### Ethics Statement

This study was conducted in compliance with the Guidelines for the Care and Welfare of Cephalopods (Fiorito et al., 2014, 2015) and the principles of the European Directive (2010/63/EU), which regulate animal research, including cephalopods, in the European Union (E 121 U; Smith et al., 2013) and, with recommendations of the ARRIVE Guideline (Kilkenny et al., 2010) for reporting *in vivo* experiments with research animals. The Institutional Animal Care and Use Committee (IACUC) of the University of Texas where this study was conducted did not require researchers to submit protocols for the ethical treatment of invertebrate larvae when this research was performed.

## Results

### Arm-Crown Complex Morphological Development During Ontogeny

#### At Hatching (2.0–2.7 mm ML; Mean TL = 1.58 ± 0.04 mm)

**FIGURE 1:**
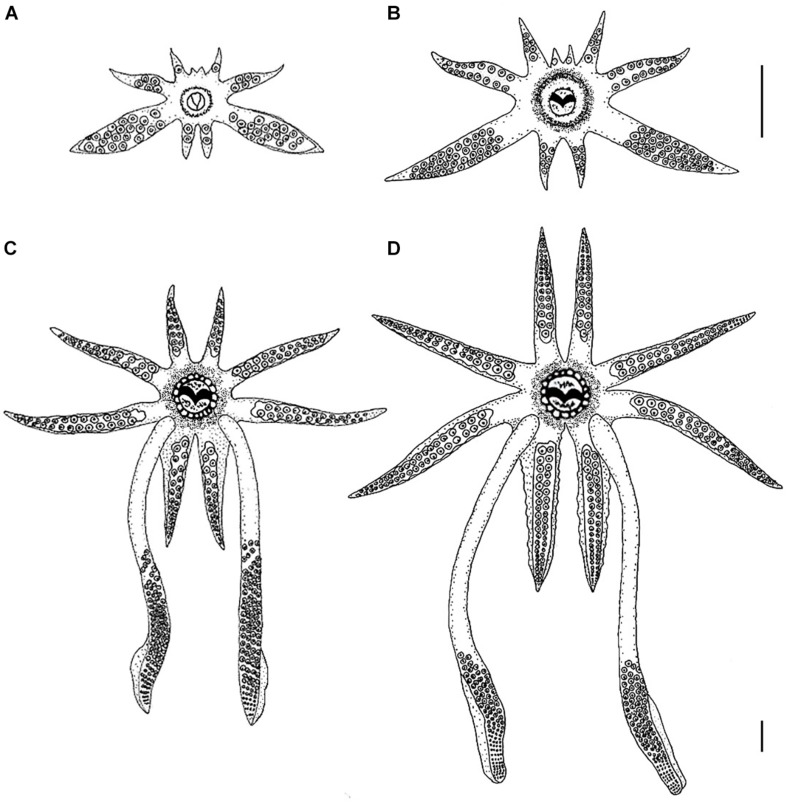
Morphological development of the arm-crown complex of *Doryteuthis opalescens* paralarvae and juveniles. **(A)** 2.7 mm ML (1 day-old); **(B)** 4.7 mm ML (30 day-old); **(C)** 10.0 mm ML (60 day-old); **(D)** 13 mm ML (80 day-old). The upper scale bar applies to panels **(A)**, **(B)**, and **(C)**; the bottom scale bar refers to panel **(D)**. Scale bars = 1 mm. Panel **(A)** is reprinted by permission from Springer: Hydrobiologia, **725**, 85–103. *Beak development of early squid paralarvae (Cephalopoda: Teuthoidea) may reflect an adaptation to a specialized feeding mode*, by R. M. Franco-Santos and E. A. G. Vidal, Copyright 2013 Springer Science + Business Media Dordrecht.

Hatchlings have a rudimentary arm crown, with arm pair I (AI) arrested at the bud stage, arm pair II (AII) has only 1 sucker, arm pair III (AIII) has about 5 suckers and the fourth pair (AIV) has 2 suckers. The arm formula is III:IV:II:I (Figure 1A). The tentacles are easily discernible from the other arms, being larger and thicker than AIII. The tentacles possess about 18–20 suckers that are distributed along their entire length. As such, the tentacles resemble an arm as neither the tentacular clubs nor the stalks are differentiated (Figure 1A). No suckers are present at the tips of the arms or tentacles.

#### 30 Day-Old (3.5–5.0 mm ML; Mean TL = 2.36 ± 0.3 mm)

The length of arms and tentacles and the number of suckers on them increased when compared to hatchlings (Figure 1B). AI has 1 sucker, AII 6, AIII 17, and AIV 7. On the arms, the suckers are distributed in two alternate rows as in the adults. The tentacles have about 38–40 suckers in four rows, covering about 80% of the tentacle’s length, the remaining 20% represents the stalks.

#### 60 Day-Old (9.5–13.0 mm ML; Mean TL = 5.8 ± 0.83 mm)

Major morphological changes occurred in the tentacles and arm-crown between 30 and 60 days of age. The tentacles have about 24 distal sucker rows which occupy approximately 50% of the tentacular length, forming a long club, clearly separated from the stalk, which represents nearly 50% of the tentacular length (Figure 1C). On the clubs, the 4 sucker rows that characterize the Genus are already present on the manus, but the carpus and dactylus are not yet differentiated. The arms show considerable increase in both length and number of suckers, particularly AI and AIV. AI has about 14 suckers, AII 24, AIII 28, and AIV 30. Swimming keels (lateral expansions) are present on the aboral surface of AIV (Figure 1C).

#### 80 Day-Old (12.0–16.0 mm ML; Mean TL = 8.7 ± 1.5 mm)

The length of the arms and their suckers increased considerably. AI has about 35 suckers, AII 42, AIII 46 and AIV 52. The tentacular clubs are well defined occupying approximately 35–40% of the tentacular length (Figure 1D). On the clubs, there are 10 rows of suckers in the manus and the dactylus is well differentiated, having about 30 rows of suckers (Figure 1D). Some modification in the relative length of the manus and dactylus will still occur before the clubs attained their final adult shape (i.e., in the adults the manus is thicker with larger suckers and the dactylus is longer and thinner (see Jereb and Roper, 2005, pp. 62).

### Tentacles Morphometry and Behavior

**FIGURE 2:**
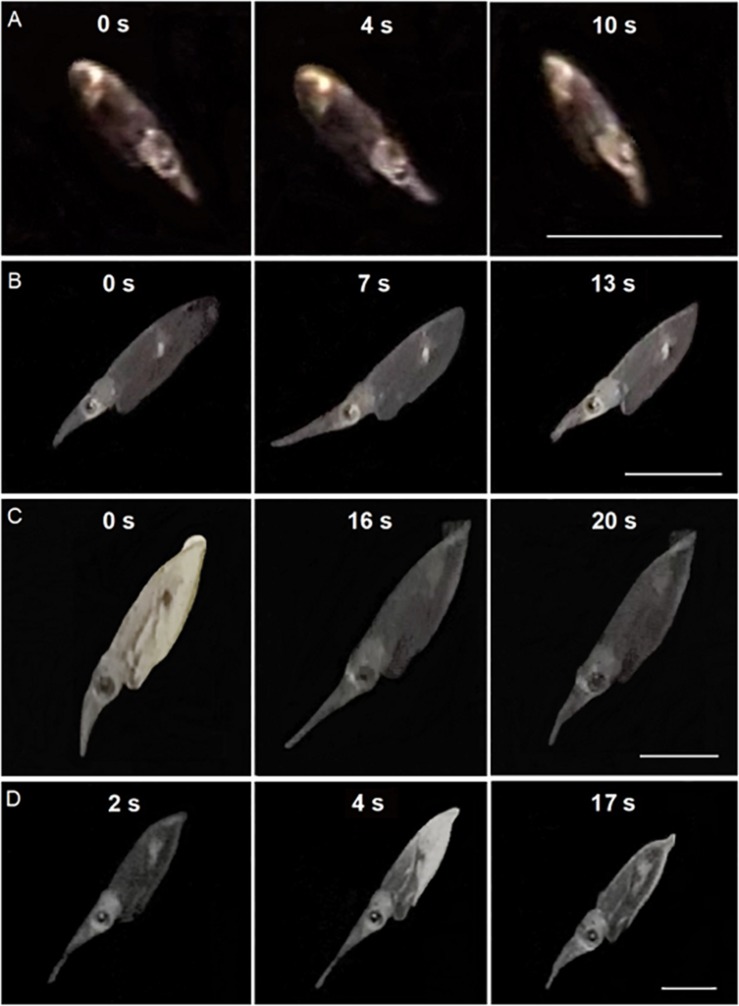
Sequential video frames of tentacular elongation and contraction cycles in *Doryteuthis opalescens*. **(A)** 14 day-old; **(B)** 40 day-old; **(C)** 50 day-old; and **(D)** 60 day-old. In sequence **(D)**, tentacles are already being elongated in the first image (2 s). Time (s) are indicated between frames. Scale bar = 5 mm.

Paralarvae were often videotaped elongating and contracting their tentacles while not attempting to capture a prey. Sequential video frames of paralarvae practicing tentacular elongations and contractions showed a progression of this movement according to size and age (Figure 2).

**FIGURE 3:**
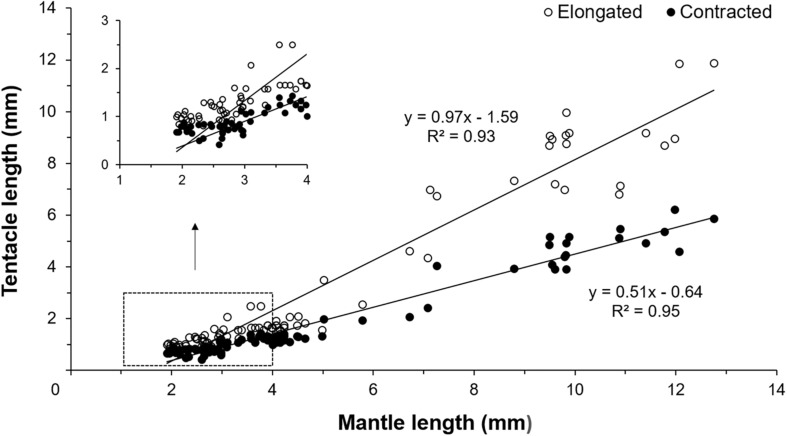
Relationship between contracted and elongated tentacles length and mantle length (ML) of *Doryteuthis opalescens* reared in the laboratory from hatching to 60 days of age (*n* = 79 contracted, *n* = 86 elongated).

Early paralarvae (<20 day-old and <4 mm ML) showed low variability between contracted and elongated TL. In contrast, in older paralarvae there was large variability and differences between contracted and elongated TL (Figures 2, 3), which resulted in significantly different regression slopes (*p* < 0.05, ANCOVA) in the relationship between contracted and elongated TL and ML (Figure 3). A wide range of elongation values were recorded for the same ML in larger squid, illustrating their enhanced ability to elongate their tentacles when compared to smaller paralarvae (Figure 3).

**FIGURE 4:**
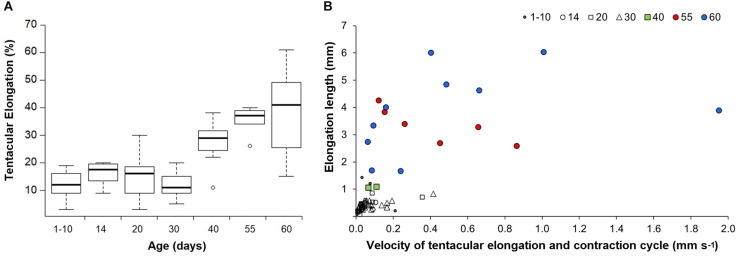
*Doryteuthis opalescens*. **(A)** Tentacular elongation percentage between contracted and elongated tentacle modes in relation to age classes. Tentacular elongation percentage was calculated by the difference between contracted and elongated tentacles; the results were normalized by the squid mantle length and converted into percentages; *n* = 6–21 for each age class. **(B)** Tentacular elongation lengths in relation to velocities for a full elongation and contraction cycle. Tentacular elongation lengths were calculated by the difference between fully contracted and elongated tentacles; *n* = 2–19 for each age class.

Paralarvae younger than 30 days were only able to perform elongations from 3 to 30% of ML (mean 13%), while older paralarvae became capable of elongating their tentacles up to 61% of ML (Figure 4A). The results from the ANOVA showed overall significant differences in proportions of tentacular elongation between paralarvae age groups [*F*(6, 75) = 19.41, *p* < 0.0001; Figure 4A]. Pairwise comparisons between groups revealed that the elongation means of 40, 55, and 60 day-old paralarvae were significantly longer than 1–10, 20, and 30 day-old paralarvae (Tukey HSD, *p* < 0.01). Significant differences were also observed between 14–55 and 14–60 age groups (*p* < 0.01).

**FIGURE 5:**
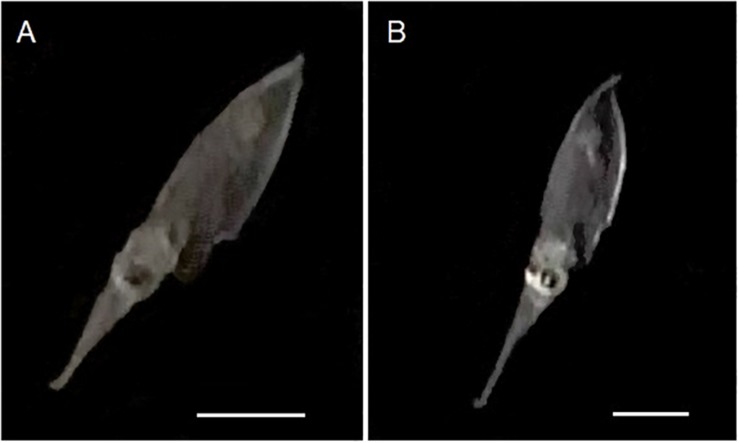
Captured images of 50 day-old *Doryteuthis opalescens* specimens bending the tips of their tentacles immediately after elongation to the maximum length and before fast contraction. **(A)** 9 mm ML; **(B)** 8.7 mm ML. Scale bars = 5 mm.

The enhanced ability of elongating the tentacles in older and larger squid was also evidenced by the velocities for a full elongation and contraction cycle. While in early paralarvae elongation lengths (difference between fully elongated and contracted tentacles) were very small, resulting in velocities <0.2 mm s^–1^, squid older than 40 days (>6.7 mm ML) became capable of longer elongation lengths (up to 6 mm), reaching velocities of up to and higher than 1.0 mm s^–1^ for a full elongating and contracting movement (Figure 4B). Additionally, paralarvae older than 40 days were observed bending the distal tip of their tentacles outward for the first time, exposing the clubs just after elongation to their maximum length and before fast contraction (Figure 5).

Significant differences between paralarvae age groups were also observed in relation to tentacular strain [*F*(6, 70) = 8.64, *p* < 0.0001]. Larger differences occurred between 60 day-old compared to 1–10 and 30 age groups (Tukey HSD, *p* < 0.001). Differences were also significant between 1–10 and 55, 20–40, 20–60, 30–40, and 30–55 age groups (*p* < 0.05). The mean (±standard error) strains measured in 1–10, 14, 20, 30, 40, 55, and 60 day-old paralarvae were 0.39 ± 0.05, 0.67 ± 0.11, 0.46 ± 0.05, 0.37 ± 0.03, 0.76 ± 0.10, 0.77 ± 0.05, and 0.87 ± 0.13, respectively.

### Predatory Behaviors

**FIGURE 6:**
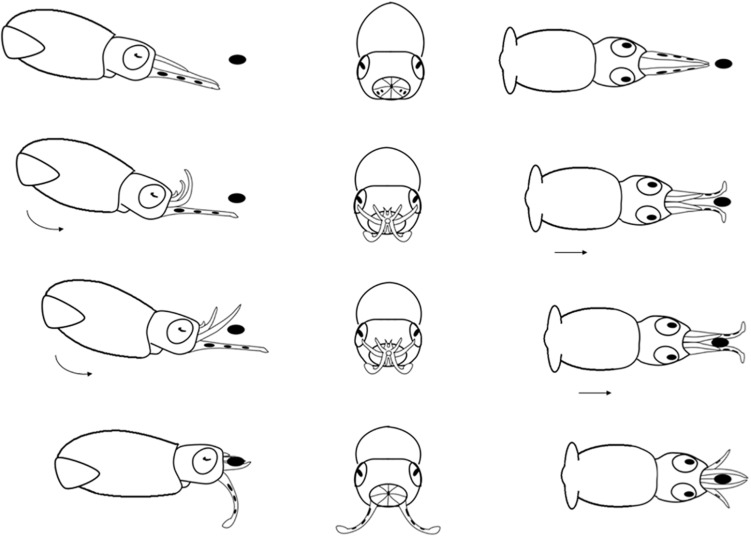
Diagrammatic sequence of the role played by the arms and tentacles of *Doryteuthis opalescens* paralarvae during the Basic Attack behavior (BA). The behavior was divided into 4 sequential stages (shown in rows) and diagrammed from three perspectives; lateral, anterior and dorsal (showed in columns). Each row represents one moment in time seen from three different angles. The dark oval represents the prey item and it was omitted from the anterior perspective because it would obfuscate the diagram. Paralarvae orient toward the prey. Their arms and tentacles are held together in a tight cone as they jet forward along a curve, positioning the arms underneath the prey. The arms spread out and the tentacles are held straight. The first contact with the prey is made with arm pairs II and III that are brought down onto the “platform” created by the tentacles. Prey is hold by the arms and the tentacles are not involved in prey holding.

**FIGURE 7:**
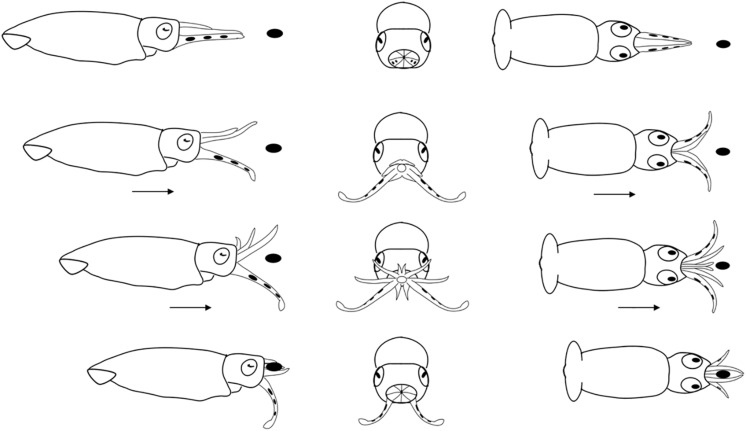
Diagrammatic sequence of the role played by the arms and tentacles of *Doryteuthis opalescens* paralarvae during the Arm-net behavior (AN). The behavior was divided into 4 sequential stages (shown in rows) and diagrammed from three perspectives; lateral, anterior and dorsal (showed in columns). Each row represents one moment in time seen from three different angles. The dark oval represents the prey item and it was omitted from the anterior perspective because it would obfuscate the diagram. Paralarvae orient toward the prey. The arms are peeled back slightly. The tentacles are ejected down and out but not directly at the prey and at the same instant the paralarvae begin a forward jet in a straight trajectory. The arms fully open as the paralarvae jet toward the prey. As the prey come into contact with the arms, they are closed and hold the prey. The tentacles play no role in prey holding.

**FIGURE 8:**
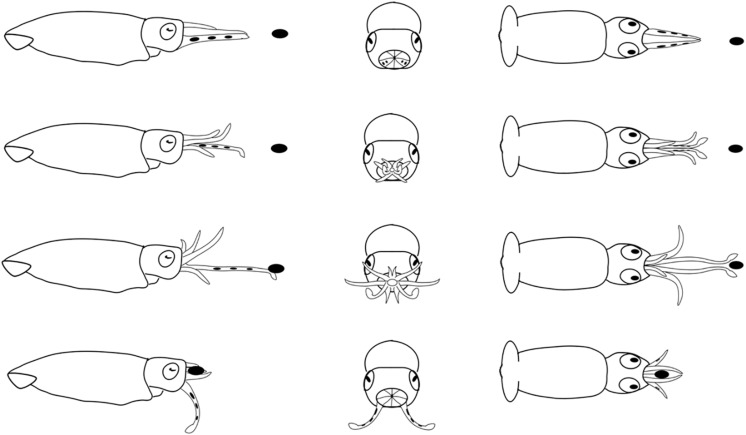
Diagrammatic sequence of the role played by the arms and tentacles of *Doryteuthis opalescens* squid during the tentaclular strike behavior (TS). The behavior was divided into 4 sequential stages (shown in rows) and diagrammed from three perspectives; lateral, anterior and dorsal (showed in columns). Each row represents one moment in time seen from three different angles. The dark oval represents the prey item and it was omitted from the anterior perspective because it would obfuscate the diagram. Squid orient toward the prey and when the prey is positioned in front of arms and tentacles, the arms begin to peel back from the tentacles prior to the strike. The tentacles are ejected forward in a straight trajectory directly at the prey. The first contact with the prey is made by the tentacular clubs. The tentacles contracted and pulled the prey into the open arms that hold the prey. The tentacles play no role in prey holding.

Three predatory behaviors were observed during the first 60 days after hatching. In general aspects, these behaviors (see below) were similar to those described by Chen et al. (1996). Here, we expanded this initial study providing other details and documenting the role played by the arms and tentacles during predatory behavior. Each predatory behavior was divided into four sequential stages (shown in rows; Figures 6–8) and diagrammed from three perspectives; lateral, anterior and dorsal (shown in columns; Figures 6–8). Each row represents one moment in time seen from three different angles (lateral, anterior and dorsal). The dark oval shape represents the prey item and it was omitted from the anterior perspective to show unobstructed views of arms and tentacles.

#### Basic Attack (BA)

This behavior is observed in hatchlings and up to 40 day-old paralarvae (2.0–6.7 mm ML; Table 1). After reacting to the prey, the paralarvae orient and move toward it until reaching an attack distance. The arms and tentacles are held together in a tight cone (Figure 6). The paralarvae begin jetting forward along a curve, with the eyes directly toward the prey and positioning its arms underneath the prey as the arms spread out to expose the suckers. The tentacles are held straight and slightly spread apart, and their distal tips are bent outward, exposing the suckers (Figure 6). Usually, the first contact with the prey is made with AII and AIII that are then brought down onto the “platform” created by the tentacles. Arm pair IV are held straight. The tentacles have suckers along their entire length, which increase the area for prey attaching (Figure 1A). Tentacles are used in the same way as the arms to hold the prey. After the arms securely grasped the prey, the tentacles hang limp and are not involved in prey manipulation. The arms play a primary role in prey holding and handling. After prey capture, the arms quickly manipulate crustacean prey (copepods, decapod zoeae, mysid shrimp) so their dorsal exoskeleton is placed in contact with the buccal mass and prey is held in this position during ingestion. The BA was not observed in paralarvae older than 40 days as it was replaced by the arm-net (AN) and the Tentacle strike.

**TABLE 1:** Comparison of the role played by the arms and tentacles during predatory behaviors in *Doryteuthis opalescens* reared in the laboratory from hatching to 60 days of age.

	**Basic attack**	**Arm-net**	**Tentacular strike**
Age (days)	0 – 40	>30	>40
Size (mm ML)	2.0 – 6.7	>4.7	>6.7
Tentacles	Not ejected	Ejected down	Ejected at the prey
Arm pairs (I, II, and III)	Fully opened	Fully opened	Fully opened
Body movement	Forward jet	Forward jet	Stationary
First contact with prey	Arms and tentacles	Arms	Tentacular clubs
Prey holding and manipulation	Arms	Arms	Arms

*ML, mantle length.*

#### Arm-Net (AN)

The AN was first observed in 30 day-old paralarvae (>4.7 mm ML) and was still present through day 60. After the paralarvae orient toward the prey, so that the prey is positioned directly in front of the cone formed by the arms and tentacles, the AI, AII and AIII are peeled back slightly. The tentacles are ejected down and out but not directly at the prey and at the same instant the paralarvae begin a forward jet in a straight trajectory (Figure 7). The dorsal arms fully open and spread apart as the paralarvae jet toward the prey, while AIV is held straight. The ejection of the tentacles downward almost in parallel orientation with arm pair AIV aids to prevent the prey from escaping underneath the dorsal arms, improving prey interception and capture. As the prey contacts the dorsal arms, they are closed and hold the prey. The AN behavior was sometimes employed by older paralarvae/early juveniles to capture motionless prey on the bottom or walls of the aquaria. The tentacles play no role in holding and handling the prey (Figure 7). Prey handling and ingestion behavior is identical to that of the BA.

#### Tentacular Strike (TS)

This behavior was first observed in 40–50 day-old squid (>6.7 mm ML). The prey is approached in a similar way as in the AN, however, the AI, AII, and AIII began to peel back from the tentacles prior to the strike, while AIV is straight, allowing the tentacles to be ejected forward in a straight trajectory directly at the prey (Figure 8). The first contact with the prey is made by the suckered surface of the tentacular clubs. By the time the tentacles reach and attach to the prey, the AI, AII, and AIII are fully opened and spread apart, while AIV is straight. During the TS, paralarvae are stationary and their position is maintained by strong fin beats. The tentacles then contract and pull the prey into the open arms that hold and manipulate the prey. As in the other two behaviors, the tentacles play no role in holding and handling the prey; the arms usually quickly flip the prey into the dorsal side prior to immobilization followed by ingestion. This was particularly noticeable with large prey, such as mysid shrimp. The prey is ingested in the same manner as in the two previous behaviors (Figure 8).

### Comparison of Predatory Behaviors

**FIGURE 9:**
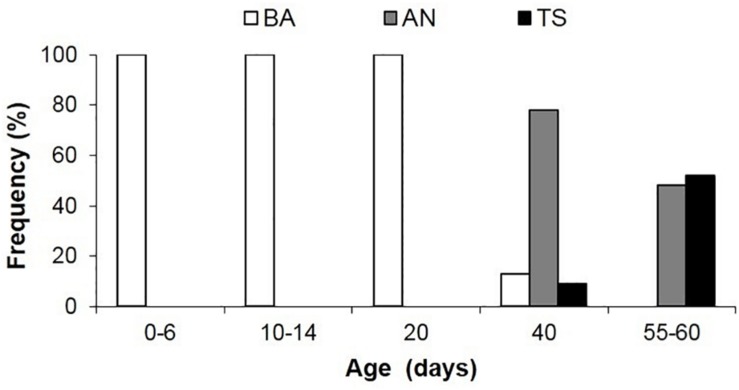
Frequency of occurrence of predatory behavior in *Doryteuthis opalescens* squid reared in the laboratory from hatching to 60 days of age. BA = Basic Attack; AN = Arm-net; TS = Tentacular strike. Age classes 0–6 (*n* = 19), 10–14 (*n* = 12), 20 (*n* = 23), 40 (*n* = 27), 55–60 (*n* = 30).

In the BA, the tentacles are used like another pair of arms during prey capture. This is the first and only feeding behavior exhibited early after hatching; it begins to wane in 30–40 day-old squid (4.7–6.7 mm ML; Figure 9 and Table 1). The BA is not observed in squid older than 40 days, when the AN is firmly developed showing the highest frequency of occurrence (Figure 9). The TS is first observed in 40 day-old squid, but with a lower frequency of occurrence (9%); however, the occurrence of this behavior increases rapidly and it is more frequent than the AN in 55–60 day-old squid (Figure 9).

The tentacles are ejected in both the AN and the TS. But in the AN the first contact with the prey is made by the dorsal arms and the tentacles are ejected downward, not directly at the prey (Table 1).

### Main Prey Types Captured by Paralarvae

**FIGURE 10:**
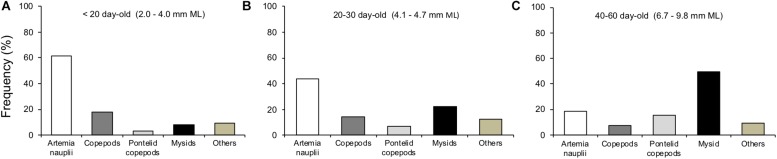
Feeding frequency of *Doryteuthis opalescens* on different prey types according to three size classes. **(A)** *n* = 42, **(B)** *n* = 32, and **(C)** *n* = 55.

The main prey types captured by paralarvae during filming were enriched *Artemia* spp. nauplii, copepods and mysid shrimp. *Artemia* spp. nauplii ranged in size from 0.3 to 0.6 mm. Copepods were composed mainly by copepodits and adults of several species. Their sizes ranged from small (0.5–1.1 mm, *Corycaeus* spp., *Euterpina acutifrons*, *Paracalanus* spp.), to medium (0.8–1.6 mm, *Acartia tonsa*, *Acartia lilljeborgi*, *Calanopia* sp., *Centropages velificatus*, *Temora turbinata*, *Temora stylifera*) and large composed by Pontellid copepods (1.5–4.0 mm, *Anomalocera ornata*, *Labidocera aestiva*, *Pontella* spp.). Mysid shrimp, *A. almyra* juveniles and adults, ranged in total size from 2 to 11 mm. Paralarvae also captured other types of prey, such as decapod crustacean zoeae, Cladocera, Cirripedia larvae, etc., which were included in the category “others” for simplification (Figure 10).

The main prey types and sizes captured by paralarvae changed during ontogeny, prey size and diversity increased with increasing squid size. The predominant prey of early paralarvae (<20 day-old, <4.0 mm ML) were *Artemia* nauplii, representing 62% of capture frequency, followed by copepods with 21% in total (Figure 10A). As paralarvae reach 20–30 day-old, capture of juvenile mysids increased to 22%, while *Artemia* nauplii became less frequent (44%) and copepods represented 21% in total (Figure 10B). For older paralarvae (>40 day-old, >6.7 mm ML), juvenile and adult mysids were the predominant prey, representing 50% of capture frequency, followed by copepods with 22% in total (Figure 10C).

## Discussion

### Tentacles Morphological Development

Herein we have shown that the tentacles of *D. opalescens* early paralarvae resemble and function as arms during predatory behavior as they do not possess clubs and stalks yet formed. Both stalks and clubs play a crucial role during the strike and their arrested development imposes a morphological constraint for the performance of the strike behavior. The stalks and clubs develop progressively as squid grow. In hatchlings, the tentacles possess suckers on their entire length and the area with suckers is non-extensible (Kier and van Leeuwen, 1997). The stalk must growth until it represents at least 50% of the tentacles’ length, as it contains the specialized transverse cross-striated muscles fibers responsible for fast elongation (Kier, 1996, 2016), and clubs need to be formed to effectively attach to prey during the strike. In fact, our results have demonstrated that the tentacles of paralarvae younger than 40 days (>6.7 mm ML) do not possess the morphological and anatomical differentiation to perform the TS, confirming and expanding previous studies (Chen et al., 1996; Kier, 1996; Kier and van Leeuwen, 1997). It turns out that while muscle fibers, stalks and clubs develop concurrently, tentacles’ muscle elongations are repeated over and over again, perhaps to adjust muscle activity pattern and its developmental process through use. It seems that flexible muscle activation patterns would be important for paralarvae to cope with the ontogenetic changes observed in the morphology and structure of the tentacles, as well as to perfect the TS performance. Our results have shown ample variability in the kinematic pattern of tentacular elongations that along with its constant repetition by paralarvae suggests that elongations could be modulated during the strike according to several cues from the prey, such as type, size, and escape response, among others. This possibility shall be a very interesting topic for future studies. For example, octopus can extend its arm to seize a prey by a wave-like propagation of a bend that travels from the base of the arm toward the extremity (Kier and Smith, 1985; Gutfreund et al., 1996). To achieve point-to-point movement, they use a quasi-articulated structure that has two bends that divide the arm into three segments, which are dynamically adjusted. The segment lengths appear the multijointed, articulated limbs of animals with rigid skeletons (Sumbre et al., 2005).

In paralarvae, results from the morphometric analysis showed intrinsically correlated changes with behavior, illustrating that early paralarvae have little or no capacity to elongate their tentacles. This ability increased with age and size and those older than 40 days (>6.7 mm ML) could elongate the tentacles up to 61% of their ML (Figure 4A). Considering that in adult loliginid squids the strike involves an elongation of 40–80% of the tentacles resting length (Kier and van Leeuwen, 1997; van Leeuwen and Kier, 1997), the elongation percentage values obtained in the present study for squid older than 40 days were quite similar. It is also interesting to notice that the mean tentacular strain values (0.46–0.87) recorded in the present study for *D. opalescens* paralarvae older than 20 days were surprisingly similar to peak strains registered for adults *Loligo pealei* (0.43–0.80; Kier and van Leeuwen, 1997). Such comparison must consider that these authors calculated strain values based on the estimated tentacular stalks length (not including the clubs), while our measurements considered the total length of the tentacles. Thus, as the clubs become smaller and the stalk portion increases relative to the size of the tentacles in older paralarvae/early juveniles, strains values would become even more comparable to those recorded from adults. On the other hand, although older paralarvae were able to perform a complete tentacular elongation and contraction cycle much faster (≥1 mm s^–1^) than younger paralarvae (Figure 4B), this is indeed a slow movement – performed while paralarvae were not attempting to capture prey – and should not be compared with the elongation velocities of adults reported by Kier and van Leeuwen (1997), which considered only the fast elongation phase until the prey was reached.

An optimal matching of tentacles structure and functional behavioral expression occurs in squid of 6.7–7.8 mm ML (40–50 day-old), when the TS behavior was observed for the first time. Simultaneously, observations of tentacular elongations within this ML window also revealed, for the first time, squid bending up the tips of their tentacles and exposing the clubs immediately after tentacles were elongated to their maximum length (Figure 5), indicating that the TS was functional.

### Correlated Arm-Crown Morphology, Predatory Behavior, and Prey Types During Early Ontogeny

Arm-crown morphology and predatory behaviors revealed close adjustments with progressive complexity and efficiency as paralarvae go through major developmental milestones. Hatchlings have a rudimentary arm-crown and fins, bell-shaped body, limited swimming abilities (Vidal et al., 2018) and display only a basic predatory behavior (BA). In this behavior, the arms and tentacles frame prey from underneath (Figure 6). These paralarvae fed mainly on small prey such as *Artemia* nauplii and copepods (nauplii, copepodits and adults), but also on other small planktonic prey, such as Crustacea brachyuran zoeae (Figure 10). Observations of prey handling by hatchlings revealed that their short arms were particularly advantageous to manipulate these preys, which have many appendages and spines (Vidal, EAG unpublished data). The underdeveloped arm-crown of hatchlings seems to make them specialized in foraging smaller prey (Figures 1A, 10). Nevertheless, paralarvae broaden their foraging repertoire by performing kleptoparasitism – feeding on prey already subdue by another squid – an imitative foraging behavior that allows several paralarvae to feed on a larger prey, such as mysid shrimp (Vidal et al., 2018).

Additionally, the ontogeny of predatory behavior of *D. opalescens* paralarvae on copepods revealed a refinement of the BA that consisted in circling copepods to find the best attack position (Chen et al., 1996). These authors have shown that through trial and error paralarvae learned to refine the BA position from posterior to anterior and approach copepods head-on, which increased the capture success.

A more elaborated behavior, the AN was first observed in 30 day-old (>4.7 mm ML) paralarvae and was depicted by the ejection of the tentacles downward (not toward the prey) with a forward jet in straight line to intercept the prey. When the arm-crown of 30 day-old paralarvae is compared with that of hatchlings, the fast development of all arms, but AI is quite conspicuous (Figures 1A,B). Although these paralarvae can eject their tentacles, this ability is still limited, as the stalk portion represents only 20% of the tentacles’ length. During the AN behavior, AIV is positioned almost in parallel orientation to the stalks (Figure 1B), which increases the area with suckers for prey interception, compensating for the lack of suckers on the stalk. This in turn should reduce the chances of prey escaping below the dorsal arms, likely improving capture success on larger prey, such as Pontellid copepods and adult mysids (Figure 10).

The AN is a transition for the TS behavior. Nevertheless, it was sometimes employed by older paralarvae/early juveniles to capture sluggishly moving or motionless prey. It is quite interesting that this predatory behavior was observed in adults *Doryteuthis pealeii* also toward slowly moving or moribund shrimp prey (Kier and van Leeuwen, 1997), in adult ommastrephid squid (Flores, 1983), and in cuttlefish (Duval et al., 1984). Certainly, there will be variations of the AN between adults and paralarvae, but once it is established at the end of the first month of life it seems to persist until adulthood. On the other hand, after the AN is expressed, the BA frequency of occurrence was reduced until it vanished completely.

The TS was first observed in 40 day-old paralarvae (>6.7 mm ML) in combination with the BA and AN, with the latter being largely predominant (Figure 9). However, in 60 day-old squid the specialized TS behavior was already more frequent than the AN. In these squid, the stalks represented at least 50% of the tentacle’s length, and the clubs were clearly defined (Figure 1C). The clubs and AIV show well-developed keels, which are expanded muscular membranes to provide better hydrodynamics and as such should play a role in the strike. Actually, it was suggested that AIV provides stability and alignment to the tentacular stalks during the entire elongation phase of the strike (Kier and van Leeuwen, 1997) and its fast development (compared to the other arms) might explain its prominent role in the strike. As paralarvae grow, the size (and weight) of the head and arm-crown complex relative to the ML increases, which might be necessary to stabilize the body and counterbalance the development of fins (Vidal et al., 2018), providing locomotion stabilization.

When the three foraging behaviors observed in the present study are compared, they revealed that the arms played the main role of prey capture from hatching to up to 40 days of age. This indicates an adaptive foraging strategy in paralarvae, as the TS is not employed in prey capture. Nonetheless, after prey was captured, the roles played by the arms were stereotyped, as they did almost the same tasks in all behaviors: hold, subdue and manipulate the prey during ingestion. Most importantly, in all three behaviors, after prey was brought to the arms, the tentacles were not involved further in prey manipulation and ingestion as happens in adults (Kier and van Leeuwen, 1997). The existence of a forward jet during the BA and AN, and the actions performed by the tentacles (not ejected, ejected downward or ejected toward the prey) (Table 1), are what truly defined each predatory behavior during early ontogeny of *D. opalescens*. The expression of these behaviors and its connection to prey-capture learning might have profound effects on the ontogeny of brain development and cognition in squid.

When the TS of a 60 day-old juvenile described in the present study is compared with that of the adults (Kier and van Leeuwen, 1997), it is clear that some modifications will still occur before the adult behavior is fully established. In juveniles, the relative length of the clubs is longer and the stalks shorter. This might influence the performance of the strike as well as the type and size of the prey that can be captured by juveniles, perhaps defining their niche occupation.

A study on the development of the beak, another essential feeding structure of cephalopods, have shown that in *D. opalescens* paralarvae the rostrum protrudes and becomes pigmented in both jaws between 30 and 50 days of age (>4.5 mm ML) (Franco-Santos and Vidal, 2014). The intensity and extent of beak pigmentation (darkening) and rostrum protrusion were suggested as having an important influence on prey selection and feeding behavior in *Octopus vulgaris* paralarvae (Hernández-García et al., 2000; Franco-Santos et al., 2014). The darkening of the chitin serves as an indication of beak hardness that together with the arm-crown morphology hint to the level of development required to feeding on prey that is hard to capture, hold on to, and dilacerate for ingestion. Therefore, it is worthy of mention that the acquisition of a robust beak in *D. opalescens* occurs coincidently just prior to the display of the TS for the first time. Squid older than 40 days (>6.7 mm ML) were able to capture larger prey such as adult mysids using the TS behavior (Figures 9, 10). Furthermore, it has been proposed that foremost ontogenetic morphological changes, such as those observed in *D. opalescens* at about 40 days of age (namely rostrum protrusion, TS behavior, school formation, see below), could be also indicative of changes in vertical distribution related to prey distribution at different depths (Karpov and Cailliet, 1979; Shea and Vecchione, 2010; Vidal et al., 2018).

Although the prey types and sizes offered to paralarvae during the present study were largely influenced by the suite of prey available during zooplankton collections and the fact that *Artemia* nauplii was offered daily due to its easy accessibility, it was possible to observe a change in the main prey type, an increase in prey size and diversity with increasing squid size (Figure 10).

### Developmental Constraints and the Performance of the Specialized TS

Our behavioral observations revealed that immediately before shooting the tentacles at the prey, squid stayed stationary holding their position against the current by means of fin movements. It is noteworthy that no forward jet was involved, contrary to observations of the TS in adults *D. pealeii*, which swim forward at velocities of 0.7–1.2 ms^–1^ as the stalks elongate (Kier and van Leeuwen, 1997). Therefore, to be able to perform the TS for the first time, squid need to be competent at holding their position against a current (sustainable swimming).

A major ecological and behavioral transition occurs in the early life history of *D. opalescens* at about 40 days of age, when they can perform sustained swimming and hover against a current (Vidal et al., 2018). The development of fins during early ontogeny has a crucial role for sustained swimming as fins act as stabilizers of the body during locomotion (Stewart et al., 2010; Vidal et al., 2018). This enhanced swimming control is a precursory stage for the formation of schools.

At this point, it should be emphasized that the TS was first observed in *D. opalescens* squid of 6.7–7.8 mm ML, this size ranges overlaps to the sizes that they start to swim in schools (see data reported in Vidal et al., 2018). This is no coincidence, as early juveniles perform sustainable swimming immediately prior to ejecting their tentacles at prey (Figure 8). This indicates that the sequential underlying factors responsible for the TS expression are complex and involves different levels of development. Besides the needed morphological development of the tentacles – involving muscle fibers, stalks and clubs – squid must have acquired swimming control. The ability to perform parallel synchronized and fine-motor control swimming with nearest neighbor squid during schooling requires advanced swimming coordination and plasticity of the neurolocomotor system, which are absent in early paralarvae (Gilly et al., 1991). In addition, binocular vision, and thus precise convergent eye movements to determine the proper distance to a prey (Budelmann and Young, 1993), is another prerequisite for the TS. It seems that several features might have evolved together in squid to design the TS. Not surprisingly, the TS expression represents a major developmental milestone and demands interaction of structures on several levels of organization.

Interestingly, in octopus paralarvae the differentiation of tissue layers, including the ganglionic structure and the majority of the musculature of the arms occurs during the planktonic paralarval phase and is maintained until the end of the animal life (Nödl et al., 2015). This seems consistent with the developmental pattern in squid paralarvae, in which ultrastructural and morphological development of the tentacles is delayed to several weeks after hatching, constraining the expression of the TS during prey capture.

### Hydrodynamic Environment and Morphological Changes of Squid During Ontogeny

Over the growth of paralarvae its hydrodynamic environment is transformed radically due to changes in morphology and size and this may impose adaptive constraints on feeding mechanisms. Hydrodynamic theory provides a basis for interpreting how growth would affect important biological processes, which in turn are dependent on the interactions of an organism with its environment (Koehl, 2000). The Reynolds number (Re) expresses the relative contribution of inertial and viscous forces to the total force acting on an animal’s body. Newly hatched squid live in an environment within intermediate Re numbers (10 < Re < 200), in which both viscous and inertial flow forces play important roles (Bartol et al., 2009; Vidal et al., 2018). However, as they grow, paralarvae need to adapt to the differing functional demands of viscous and inertial regimes.

Due to the viscous drag production it was suggested that it would be easier for a hatchling to move its entire bell-shaped body (predicted to generate relatively low drag) to seize a prey instead of shooting the tentacles (Vecchione, 1987). Indeed, this is exactly what we have shown during the BA and AN behaviors (Figures 6, 7). Even if the tentacles of hatchlings would be morphologically functional to be eject during prey capture, hatchlings would not be able to hold their position against a current prior to shooting their tentacles at prey, because they are passive drifters. The precision of the TS involves swimming control. This might partially explain why there is a forward jet to intercept the prey in the BA and AN behaviors. As emphasize previously, the TS is only employed after *D. opalescens* can perform sustainable swimming and also attain a size (>6 mm ML) that afford them to transition occasionally to the inertia dominated realm (reaching high Re values), making the transition from plankton to nekton just after its first month of life (Vidal et al., 2018). Interestingly, the hatchlings of other squid families, such as Onychoteuthidae and Cranchiidae also hatch out with short tentacles, without differentiated stalks and clubs (Sweeney et al., 1992), which resemble arms as in loliginid squid. Based on the results of the present study, one can predict that these hatchlings will most likely capture their prey with a predatory behavior similar to the BA described here, involving a forward jet and an arm-strike in which the tentacles function as arms.

Overall, prey size in loliginid squid hatchlings seems to be limited by their rudimentary arm-crown and poor morphological development. It was suggested that the tentacles have evolved from modification of the AIV (Boletzky, 1993; Kier and van Leeuwen, 1997), but in hatchlings they still function as arms because of morphological constraints that impose displacement of the TS to later ontogenetic stages. This in turn called for a change in feeding strategy toward smaller prey and an adaptative predatory behavior (BA) allowing hatchlings to explore an ecological niche different from the adults. Ultimately, there is a trade-off between the poor morphological development of loliginid squid hatchlings and their exponential growth rates. The latter appears to be a selection on paralarvae to reach the adult morphology as rapidly as possible.

## Conclusion

This study documented the ontogeny of the predatory behavior in *D. opalescens* paralarvae as they undergo critical developmental milestone. Our results were combined with available literature to demonstrate that arm-crown morphology, swimming abilities and predatory behaviors of paralarvae show interdependency and progressive complexity during ontogeny. Hatchlings have overall poor morphological development, limited swimming abilities and little or no capacity to elongate their tentacles. The tentacles of hatchlings resemble and function as arms during early predatory behavior (BA), as the stalks and clubs are not yet formed. Over the first month of life, the ability to eject the tentacles develops progressively as paralarvae experience major changes in body form, swimming performance, and arm-crown morphology and structure. Paralarvae were often observed elongating and contracting their tentacles, while not attempting to capture prey, suggesting that this behavior could perhaps serve to adjust muscle activity and its developmental process through use, while specializations for the strike (stalks, clubs, muscle fibers, arm-crown complex) are not yet fully formed.

When paralarvae reach 30 days of age, the AN behavior was firmly in place and prevailing over the others. This predatory behavior represents a transition from the BA to the TS and continues to be employed by adults. Squid older than 40 days (>6.7 mm ML) became capable of performing tentacular elongations significantly larger (up to 61% of the ML) and a complete tentacular elongation and contraction cycle much faster (≥1 mm s^–1^) than the younger ones. An optimal coordination between tentacles structure and functional behavior happened in squid of 40–50 days of age (6.7–7.8 mm ML), when clubs and stalks were formed, and squid were observed bending up the tips of the tentacles to expose the clubs immediately after fully elongations. This event also coincided with the expression of the TS behavior for the first time.

The TS was first observed in *D. opalescens* simultaneously with their ability to swim in schools (see Vidal et al., 2018), as swimming control is a prerequisite for the performance of the strike in early juveniles. This emphasizes that the sequential underlying factors responsible for the TS expression are complex and involves different levels of development: muscle fibers, stalks and clubs differentiation, arm-crown (particularly arm IV), swimming coordination (schooling) and binocular fixation of the prey.

The arms played the main role of prey capture in squid younger than 40 day-old (<6.7 mm ML) as the TS was not functional. After prey was captured, the roles played by the arms were stereotyped, as they did almost the same tasks in all behaviors: hold, subdue and manipulate the prey during ingestion. On the contrary, the actions played by the tentacles were what really defined each predatory behavior during squid early ontogeny. However, after prey was brought to the arms, the tentacles were not involved further in prey manipulation and ingestion as happens in adults. The predatory behaviors of paralarvae/early juveniles are adapted to the functional demands imposed by the developing morphology, structure and mechanics of the tentacles, as well as swimming coordination (Vidal et al., 2018).

By correlating behavioral observations with morphological and morphometric data, this study documented interconnected morphological and behavior traits that enabled squid to perform the TS, offering new insights into the interdependency of morphology and predatory behaviors during squid ontogeny.

## Data Availability Statement

The datasets generated for this study are available on request to the corresponding author.

## Ethics Statement

Ethical review and approval was not required for the animal study because The Institutional Animal Care and Use Committee (IACUC) of the University where this study was conducted did not require researchers to submit protocols for the ethical treatment of invertebrate larvae when this research was performed.

## Author Contributions

EV conceived and designed the study, conducted the experiments, collected and analyzed the data, and drafted the manuscript. BS helped with the data analysis, visualization, preparations, and made improvements to the manuscript. Both authors worked together to interpret the findings and approved the final version.

## Conflict of Interest

The authors declare that the research was conducted in the absence of any commercial or financial relationships that could be construed as a potential conflict of interest.
